# Rapid Physicochemical Changes in Microplastic Induced by Biofilm Formation

**DOI:** 10.3389/fbioe.2020.00205

**Published:** 2020-03-20

**Authors:** Eric McGivney, Linnea Cederholm, Andreas Barth, Minna Hakkarainen, Evelyne Hamacher-Barth, Martin Ogonowski, Elena Gorokhova

**Affiliations:** ^1^Department of Environmental Science, Stockholm University, Stockholm, Sweden; ^2^Department of Fibre and Polymer Technology, KTH Royal Institute of Technology, Stockholm, Sweden; ^3^Department of Biochemistry and Biophysics, Stockholm University, Stockholm, Sweden

**Keywords:** microplastic, biofilm, biodegradation, microbiome composition, physicochemical characterization, polyethylene, polypropylene, polystyrene

## Abstract

Risk assessment of microplastic (MP) pollution requires understanding biodegradation processes and related changes in polymer properties. In the environment, there are two-way interactions between the MP properties and biofilm communities: (i) microorganisms may prefer some surfaces, and (ii) MP surface properties change during the colonization and weathering. In a 2-week experiment, we studied these interactions using three model plastic beads (polyethylene [PE], polypropylene [PP], and polystyrene [PS]) exposed to ambient bacterioplankton assemblage from the Baltic Sea; the control beads were exposed to bacteria-free water. For each polymer, the physicochemical properties (compression, crystallinity, surface chemistry, hydrophobicity, and surface topography) were compared before and after exposure under controlled laboratory conditions. Furthermore, we characterized the bacterial communities on the MP surfaces using 16S rRNA gene sequencing and correlated community diversity to the physicochemical properties of the MP. Significant changes in PE crystallinity, PP stiffness, and PS maximum compression were observed as a result of exposure to bacteria. Moreover, there were significant correlations between bacterial diversity and some physicochemical characteristics (crystallinity, stiffness, and surface roughness). These changes coincided with variation in the relative abundance of unique OTUs, mostly related to the PE samples having significantly higher contribution of *Sphingobium, Novosphingobium*, and uncultured Planctomycetaceae compared to the other test materials, whereas PP and PS samples had significantly higher abundance of Sphingobacteriales and Alphaproteobacteria, indicating possible involvement of these taxa in the initial biodegradation steps. Our findings demonstrate measurable signs of MP weathering under short-term exposure to environmentally relevant microbial communities at conditions resembling those in the water column. A systematic approach for the characterization of the biodegrading capacity in different systems will improve the risk assessment of plastic litter in aquatic environments.

## Introduction

Plastic pollution is a growing problem. Much of the plastic litter found in our oceans are small fragments; a class of pollutants known as microplastic (MP; operationally defined as particles <5 mm in diameter). The small size, ubiquity, and persistence have caused MP to become a global concern and a focus of environmental research.

Once plastic litter enters the environment, it is subjected to physical, biological, and chemical forces leading to its transformation. In turn, this transformation affects transport, bioavailability, and fate of MP (Galloway et al., 2017; Botterell et al., 2019). Physical changes include aggregation and sorption of macromolecules, as well as mechanical abrasion of the polymer surfaces, whereas chemical transformations of MP include, e.g., photo-oxidation and hydrolysis, which can lead to chain scission and changes in the physical integrity of the polymer. Biological transformations occur in concert with physical and chemical changes and include degradation and oxidation of the polymer by microorganisms living in association with polymer surface in a complex multilayer community. The nature of MP transformations needs to be studied to understand the environmental risks posed by these emerging contaminants.

In the aquatic environment, microbial colonization of marine litter is a quick process. Initial colonization of microplastics by environmental microorganisms occurs within minutes to hours (Harrison et al., 2014). Not only do biofilms thrive on polymer surfaces, but they often differ from the ambient communities or biofilms found on natural substrates in the same habitats (Zettler et al., 2013; Harrison et al., 2014; Bryant et al., 2016; Kettner et al., 2017; Dussud et al., 2018; Ogonowski et al., 2018). Also, as with any other ecological communities, the MP-associated microbial communities vary depending on the season, environmental factors, and location (Oberbeckmann et al., 2014). The taxonomic differences induced by selectivity toward specific substrates often imply differences in functional properties and metabolic rates (Philippot et al., 2010). In line with this, Bryant et al. (2016) found an increase in metabolic and biogeochemical activity in plastic-associated biofilms compared to the ambient community, manifested by the increase in oxygen production and expression of genes responsible for secretion systems, chemotaxis, cell-to-cell interactions, and nitrogen fixation.

Biodegradation of polymers has long been studied (Shah et al., 2008), but our understanding of the relationships between polymer properties and microbial assemblages associated with the polymer surfaces is still limited (Urbanek et al., 2018). Several studies have identified hydrocarbon-degrading bacteria colonizing plastic debris in seawater (Reisser et al., 2014; Ogonowski et al., 2018; Urbanek et al., 2018). Microbial degradation of polymers has even been proposed as a potential remediation practice to eliminate plastic waste from ecosystems (Pathak and Navneet, 2017; Urbanek et al., 2018). A recent study suggested that dissolved organic carbon released from plastic pollution in the oceans is altering the biogeochemical fluxes and microbial landscape in the marine environment (Romera-Castillo et al., 2018).

Some studies have also implicated microbial colonization as a driver of MP surface transformations. Zettler et al. (2013) observed pits carved into the polymer surfaces that conformed to the shape of the bacterium that resided in it, suggesting polymer degradation (Zettler et al., 2013). Other physicochemical properties of the polymers that have been shown to change after microbial colonization include crystallinity (Santo et al., 2013), mass loss (Tribedi and Sil, 2012), hydrophobicity (Wang et al., 2011), molecular weight (Santo et al., 2013), topography (Webb et al., 2009; Nowak et al., 2011; Zettler et al., 2013; Reisser et al., 2014), and surface functionalization (Tribedi and Sil, 2012).

Although several studies reported that biofilm communities growing on plastic litter in marine systems are different from those on natural substrates (Zettler et al., 2013; Harrison et al., 2014; Bryant et al., 2016; Kettner et al., 2017; Dussud et al., 2018; Ogonowski et al., 2018), others found no such differences (Witt et al., 2011; Oberbeckmann et al., 2016). Most importantly, very few studies have related biofilm community structure to the physicochemical properties of the substrate. Recently, we showed that bacterioplankton from the Baltic Sea exposed to MP (polyethylene [PE], polypropylene [PP], and polystyrene [PS]) displayed lower community diversity and evenness compared to the source community, but also to the biofilms developing on cellulose and glass particle controls in the same environment, suggesting substrate-driven selection (Ogonowski et al., 2018). Interestingly, variation in the community structure was linked to the substrate's theoretical hydrophobicity (Ogonowski et al., 2018); however, no physicochemical MP characterization was considered in this study.

Current research on plastic litter and its environmental fate lacks a connection between the composition and functionality of biofilms, on the one hand, and MP behavior and transformation on the other hand. There is a need to study MP-biofilm interactions in environmentally relevant conditions and to investigate how microbial colonization alters MP properties, but also how these properties may promote colonization and composition of communities. The aims of our study were, therefore, to test (1) whether physicochemical properties of MP from commercially important polymers change during biofilm formation, (2) if a diversity of the biofilm developing on the MP is related to their physicochemical properties, and (3) what microorganisms are associated with these changes.

## Methods

We capitalized on the material collected as a part of the experimental study reported elsewhere (Ogonowski et al., 2018). To evaluate whether the physicochemical properties of MP were affected by biofilm formation, we conducted a comprehensive physicochemical characterization of MP beads exposed to the bacterioplankton from the Baltic Sea as described in section Exposure Experiments. We also conducted an additional experiment to control for any effects of bacteria-free water on the polymers during the exposure. The physicochemical properties characterized in MP before and after the incubation included stiffness, maximum compression, crystallinity, surface chemistry, and topography. Moreover, we reanalyzed the primary sequencing data on bacterial in the biofilms to address the linkages between the biofilm diversity and the polymer properties and to identify taxonomic groups of the microorganisms associated with the physicochemical changes.

### Microplastics

Spherical beads of polyethylene (PE), polypropylene (PP), and polystyrene (PS) purchased from Cospheric LLC (Santa Barbara, CA; www.cospheric.com) were used as model MP (Supplementary Table 1). The MP were characterized previously in terms of primary particle size, density, specific surface area, and nominal values for surface hydrophobicity (Ogonowski et al., 2018). The particles have no coating, but manufacturer provides no information on the additives used for the polymer synthesis; it is stated, however, that the material contains no hazardous substances.

### Exposure Experiments

The experimental MP beads were exposed to: (1) ambient bacterioplankton forming a biofilm on the bead surface (referred to as *Biofilm* hereafter) and (2) sterile water, where no biofilm was present (referred to as *Water* hereafter). Untreated MP were used as *Controls* in both treatments (*Control Biofilm* and *Control Water*, respectively). The methods for *Biofilm* treatment are described in detail elsewhere (Ogonowski et al., 2018). Briefly, brackish water (3.5 PSU) was collected from a coastal bay in the northern Baltic proper, Sweden, in August 2014 (59°23′2.14″N, 18°10′50.17″E). Before the experiment, potential bacterivores were removed from the source water by filtering through a 6-μm sieve. Each polymer was then incubated in 1 L of the filtered seawater under controlled light conditions (16:8 h of light/dark) and room temperature (22°C). The incubation vessels were gently stirred three times per week for 2–3 min. After the 2-week incubation, the MP beads were recovered from the experimental vessels and split in two batches. One batch was used for DNA extraction and characterization of the bacterial community by 16S rRNA gene sequencing as described previously (Ogonowski et al., 2018), whereas the other batch was stored at −20°C for approximately 2 years. The *Water* treatment involved incubating MP under the same light and temperature conditions and during the same period. All treated MP and their respective controls were subjected to physical and chemical analyses as described below.

### Compression Testing

Compression tests were used to measure the change in brittleness of the MP beads after the biofilm formation. The analysis was conducted with Instron 5,944 tensile testing machine with a compression rate of 0.1 mm/s; PE was tested with a 50 N load cell, whereas PP and PS were tested with a 500 N load cell. All samples were conditioned for 48 h in the testing room before the measurements (22°C, 40% relative humidity). Maximum compression, ε_*max*_, a measure of how much force a material can withstand before it breaks, was calculated from the initial diameter, $\bar{d}$ (mm), and deformation diameter, *D* (mm) as:

(1) $$\begin{array}{r} \varepsilon_{max}=\frac{D}{\bar{d}} \end{array}$$

Initial diameter measurements were done for each particle (*n* = 9–12) at randomly selected orientations.

The stiffness (*k*, N/mm) of a material is a measure of how much force it can withstand before it becomes deformed. It was calculated using a linear region of the load strain curve and presented as an average of three measurements for each sample:

(2) $$\begin{array}{r} k=\frac{\Delta F}{\Delta \epsilon_{comp}} \end{array}$$

Where Δ*F* (N) is the change in force, and Δε_*comp*_ (mm/mm) is the change in compression.

### Degree of Crystallinity

The MP beads crushed in the compression test were used to measure the degree of crystallinity by differential scanning calorimetry (DSC, Mettler/Toledo) with a sample robot and cryo-cooler. The crushed samples (4–5 mg/sample) were used instead of intact MP to increase the contact area with the DSC cup, which gives more accurate values. The heating for PE and PS was 25–190°C and 25–150°C, respectively, starting with a cycle of heating and cooling, followed by a second heating. For PP, the program started with a cooling from 25 to −30°C, followed by heating/cooling/heating ranged between −30 and 220°C. For all samples, the heating/cooling rate was 10°C/min, with an isothermal period of 1 min between each dynamic segment (Supplementary Figure 1). All experiments were conducted under a nitrogen gas flow of 50 mL/min. The data were evaluated using Mettler Toledo STARe software (V15.00.a). The degree of crystallinity, *X*_*c*_ (%), was calculated as:

(3) $$\begin{array}{r} X_{c}=\frac{\Delta H_{m}}{\Delta H_{m}^{o}}\cdot 100 \end{array}$$

Where Δ*H*_*m*_ (J/g) is the heat of melting calculated from the integral of the melting peak and $\Delta H_{m}^{o}$ (J/g) is the theoretical heat of melting of a 100% crystalline polymer. As the theoretical heat of melting, 293 J/g was used for PE and 207 J/g for PP (Wunderlich, 1990). PS was considered completely amorphous (Wunderlich, 1990).

### Surface Topography

The surface morphology of MP beads from the *Biofilm* treatment was investigated using scanning electron microscopy (SEM) and atomic force microscopy (AFM). No samples from *Water* treatment were analyzed because AFM analysis of *Biofilm* treatment showed no significant differences between the samples before (*Control Biofilm*) and after (*Biofilm*) exposure for any MP; thus, the examination of samples from *Water* treatment and the respective controls was irrelevant to our research objectives.

#### Scanning Electron Microscopy

SEM data were collected using an ultra-high resolution FE-SEM Hitachi S-4800 SEM, at 1–5 kV. The beads were mounted onto the sample plate with carbon tape and sputter-coated for 10 s with Pt:Pd. The samples were taken out of the freezer at least 7 days before the analysis, dried and stored in a desiccator. For each sample, two MP beads were visualized.

#### Atomic Force Microscopy

The AFM measurements were performed to evaluate the surface roughness of MP. The topographical imaging was performed in ScanAsyst mode on a Bruker Multimode 8 with Nanoscope V controller and E scanner. The cantilever was made out of Si and had a spring constant of 5 N/m. The roughness was calculated by software NanoScope Analysis 1.6 following second-order flattening. The MP samples were glued to a metal plate and the upper surface was analyzed. For each material and treatment/control, two beads were analyzed, and images taken on three randomly selected spots on the surface of each sample were used for the calculations. Both the arithmetic, *R*_*a*_, and root mean squared, *R*_*q*_, roughness (nm) were calculated based on the height (*y*), position (*i*), and length (*L*):

(4) $$\begin{array}{r} R_{a}=\frac{1}{L}\Sigma |y_{i}| \end{array}$$

(5) $$\begin{array}{r} R_{q}=\sqrt{\frac{1}{L}\Sigma y_{i}^{2}} \end{array}$$

### Surface Chemistry

Attenuated total reflection-Fourier transform infrared spectroscopy (ATR-FTIR) analysis was conducted to analyze changes in functional groups on the MP surfaces. The infrared spectra were recorded at a resolution of 4 cm^−1^ on a Bruker Vertex 70 FTIR spectrometer equipped with a deuterated L-alanine-doped triglycine sulfate (DLATGS) detector and 200 interferometer scans were averaged for each spectrum. Samples were pressed on a diamond crystal Bruker Platinum attenuated total reflection (ATR) setup with the help of a piston. This technique is surface sensitive, because the infrared beam decays exponentially within the sample. The penetration depth for our setup was 0.5 μm at 4,000 cm^−1^ and 5 μm at 400 cm^−1^. For each treatment, four to eight spectra were recorded (three in the case of *PE Water*) from different surface areas of one to three beads and averaged. Although the spectrometer was continuously purged to remove water vapor and CO_2_, residual CO_2_ signals were present in the spectra and were subtracted using a spectrum that contained the CO_2_ bands in the 2,390–2,280, and 702–623 cm^−1^ regions and straight lines otherwise. No baseline correction was applied.

To emphasize the spectral changes that were induced by incubation with (*Biofilm*) and without bacterioplankton (*Water*), the respective *Control* spectra were subtracted from the *Water* and the *Biofilm* spectra for each polymer. The *Control* spectra were multiplied with an appropriate factor so that the polymer bands in the 1,500–1,350 cm^−1^ region canceled in the subtraction. Note that there is no single factor that eliminates the polymer bands in the entire spectrum. Therefore, the difference spectra may contain positive bands due to incomplete subtraction and negative bands due to over-subtraction of polymer bands outside the 1,500–1,350 cm^−1^ region.

### 16S rRNA Gene Sequencing

Bacterial DNA was extracted from the MP beads, sequenced, and processed as described previously (Ogonowski et al., 2018). Briefly, PCR amplicon libraries of the 16S rRNA encoding gene were produced using a barcoded primer set adapted for the Illumina MiSeq, TruSeq DNA PCR-free LT Library Preparation Kit (Illumina) and targeting the V3 and V4 regions of the gene. Quality control was performed on an Agilent 2100 BioAnalyser using high sensitivity DNA chip. PhiX DNA (10%) was added to the denatured pools and sequencing was performed on an Illumina MiSeq (600-cycles). DNA sequence data were generated using paired-end sequencing, demultiplexed, and subjected to quality filtering, dereplication, sorting, OTU clustering and mapping according to the UPARSE pipeline guidelines on the UPPMAX cluster (https://wiki.bils.se/wiki/Running_the_Uparse_pipeline_at_the_UPPMAX_cluster). Taxonomic identification was performed using SINA to annotate OTU clusters against the SSU NR99 SILVA database. The sequenced data were deposited through NBCI SRA project PRJNA382770 and used here.

### Statistical Analyses

#### Differences in Physicochemical Properties Induced by Exposure

Beads from the *Biofilm* and *Water* treatments were compared to their respective controls with regard to each physicochemical variable, i.e., degree of crystallinity (*X*_*c*_), stiffness (*k*), maximum compression (ε_*max*_), surface roughness (*R*_*a*_), and diameter (*d*). *F*-test for equality of variances was applied to evaluate the differences in within-group variability. As number of replicates varied among the polymers and treatments and normality tests were not always meaningful due to the low sample size, we used a two-sample bootstrap hypothesis test for difference of means when making pair-wise comparisons between the treated (i.e., after exposure) and control (i.e., before exposure) samples for each polymer type, variable, and treatment (*Biofilm* and *Water*). Non-parametric bootstrapping (10 000 bootstraps) with replacement and *n* as the number of empirical observations (3–6, depending on the variable) was applied for each *treatment—control* comparison, and the difference between the bootstrapped means of the treatment and control groups and the associated 95% confidence interval (CI) were calculated and plotted for each variable and polymer. As zero value for the difference implies no difference between the means, the difference was considered statistically significant when CI excluded zero value and the associated *p*-value, which agrees that the CI does not include zero, was <0.05.

#### Correlations Between Physicochemical Variables

For each treatment, cross-correlations between the physicochemical variables were calculated. Since the different physicochemical variables were not measured on the same MP beads (i.e., the observations were unpaired), we used Monte Carlo simulated data to account for the observed variability and to pair the data. For each variable, polymer type (PE, PP, and PS), and treatment (*Biofilm, Water*, and their respective controls), the means and corresponding standard deviations were generated and used to simulate new data sets by random pairing (*n* = 3 to keep the sample effort comparable across the treatments). Using the Monte Carlo simulated data, bootstrap resampling (*n* = 9; 10 000 resampling with replacement) was used to calculate Pearson's *r* and the associated 95% confidence intervals for each pair of the physicochemical variables within a treatment.

#### Relationships Between Physicochemical Properties and Microbial Communities

To assess the alpha diversity of the bacterial communities developed on the MP in *Biofilm* treatment, we calculated commonly used indices (Fisher's alpha, Chao1estimator, Abundance-based Coverage Estimator [ACE], and Shannon-Wiener index) that consider different properties of a community, e.g., richness and evenness as well as the relative abundance of rare taxa (Supplementary Table 2). The indices were calculated using *vegan* v2.3-5 R package, and individual sample data rarefied to equal sequencing depth at the treatment level; rarefaction was based on 999 permutations. To test whether a diversity index differed between the polymers, one-way ANOVA followed by Tukey HSD multiple comparisons was applied. All models were checked for normal distribution of residuals by examining the QQ plots.

Further, to relate the diversity metrics to the measured physicochemical properties of the polymers, correlation coefficients were calculated between the diversity indices and the mean value for each physicochemical parameter, paired by polymer type. For the pairwise correlations. Spearman's ρ was applied (α = 0.05).

#### Differential Abundance Analysis in Biofilms

To compare the differences in taxonomic composition and to assess whether some bacterial taxa were differentially abundant on specific polymers, we conducted differential abundance analysis using the rarefied OTU data, a Zero-Inflated Gaussian Distribution Mixture Model with *fitZig* function native to the *metagenomeSeq* v1.10.0 R package (Paulson et al., 2013). The differential abundance in features of the biofilms was estimated after normalizing the data by cumulative sum scaling (CSS). The function *calculateEffectiveSamples* was applied to the filtered OTU table and features with less than the average number of effective samples in all features were removed. With the coefficients from the model, we applied moderated *t*-tests between accessions using the *makeContrasts* and *eBayes* commands retrieved from the R package *limma* (v.3.22.7). Obtained *p*-values were adjusted using the Benjamini–Hochberg correction and False Discovery Rate (FDR); differences in the relative abundance of OTU between the groups were considered significant when adjusted *p*-values were lower than 0.05.

## Results and Discussion

### Exposure Effects on Physicochemical Properties of MP

**Figure 1:**
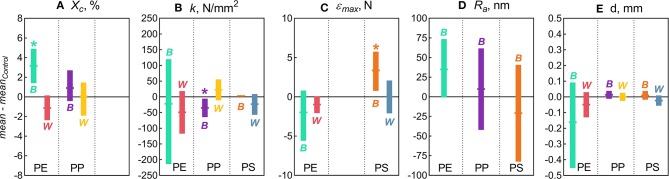
Changes in the physicochemical characteristics of the microplastic measured in this study: **(A)** Degree of crystallinity [*X*_*c*_]; **(B)** Stiffness [*k*]; **(C)** Maximum compression [ε_*max*_]; **(D)** Arithmetic roughness [*R*_*a*_]; and **(E)** Diameter [*d*]. The data are presented as mean (horizontal notches) and 95% confidence interval (vertical bars) values of the bootstrapped differences between the treatment means (*B* for *Biofilm* and *W* for *Water*) and their respective controls. Asterisks (*) indicate significant difference between the treatment and the control indicated by the distributions with the confidence interval excluding zero. No statistical comparisons were possible for crystallinity in PS because this material is amorphous (*X*_*c*_ ≈ 0).

The following properties were significantly affected by the *Biofilm* treatment: degree of crystallinity in PE, stiffness in PP, and maximum compression in PS, whereas *Water* treatment had not induced any measurable changes in any of the polymers tested (Figure 1). In addition, the variance for the particle diameter for PE and PS in *Biofilm* treatment significantly decreased compared to the controls (Figure 1E). These significant effects are presented and discussed below. As no significant changes were observed in surface roughness (Figure 1D) and other topography features in any of the polymers tested, these results are presented as Supplementary Figures 2, 4.

#### Degree of Crystallinity

**Figure 2:**
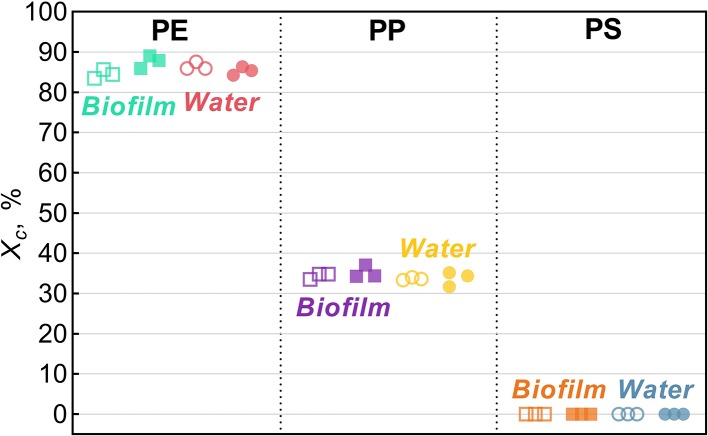
Degree of crystallinity, *X*_*c*_, for *Biofilm* (solid squares) and *Water* (solid circles) treatments and their respective untreated controls (hollow symbols). The data points represent replicate samples (*n* = 3). The polymers tested were polyethylene (PE), polypropylene (PP), and polystyrene.

Across all treatments, PE had the highest degree of crystallinity, followed by PP (*X*_*c*_ >82%; Figure 2). For PS, *X*_*c*_ was assumed to approach zero because only a small difference in *T*_*g*_ could be observed in DSC heat cycling (Supplementary Figure 1), which is not surprising as PS in its atactic form is considered amorphous (Boyer, 2007).

In the PE beads covered with biofilm, *X*_*c*_ increased significantly (by 3.2% on average) compared to the controls (two-sample bootstrap test; *p* < 0.03; Figure 1A). By contrast, no significant change in *X*_*c*_ was induced by bacteria-free water (*p* > 0.15), although in theory, this exposure might cause secondary crystallization due to increased chain mobility. Therefore, the observed *X*_*c*_ increase in PE *Biofilm* is likely due to the biological activity of the microbial communities. It is known that polymer biodegradation usually begins in the amorphous regions (Raghavan and Torma, 1992; Zuchowska et al., 1999). Another possible mechanism for the increased crystallinity in PE *Biofilm* is that the biofilm facilitated the removal of additives or other low molecular weight components that were used as a substrate or migrated from the polymer matrix to the microbial cells. The loss of these compounds could have contributed to the increased polymer crystallization. The observed increase in crystallinity after only 2 weeks of exposure to the natural bacterial community contradicts the results of a previous 3 year aging experiment, in which PE was incubated in natural seawater but no change in crystallinity was observed (Brandon et al., 2016). However, the latter study addressed the long-term transformations of MP, with the first measurement taken after 5 months of exposure; therefore, the changes occurring within weeks could not have been detected.

#### Particle Diameter

**Figure 3:**
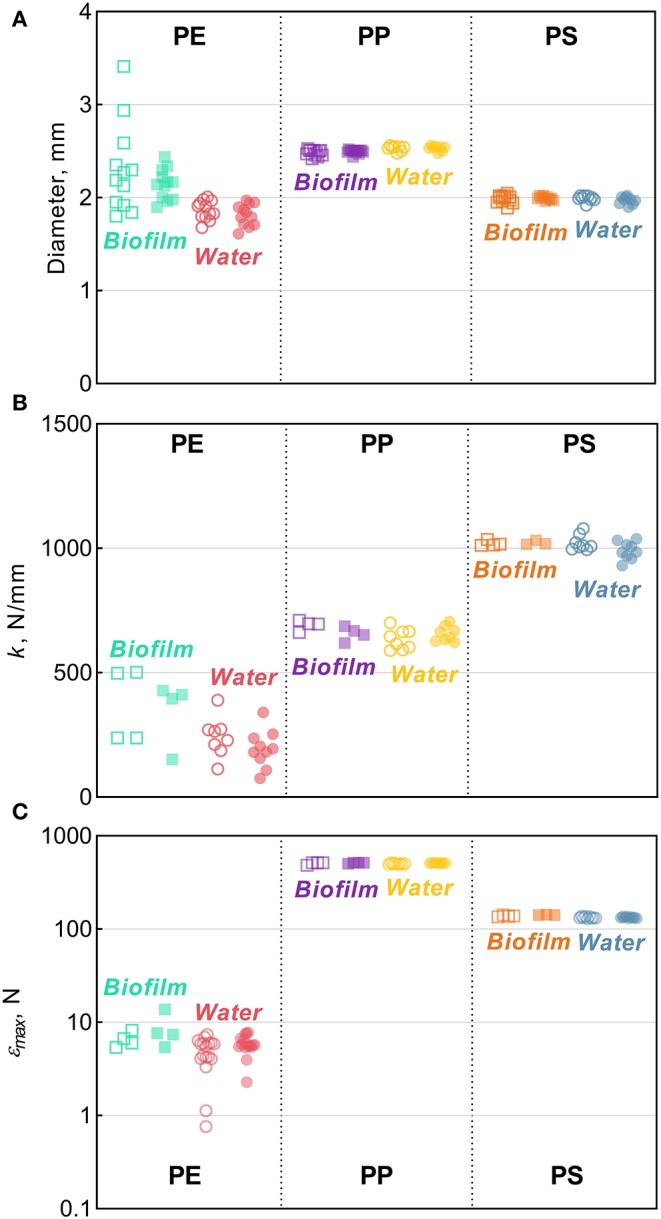
Tensile comparisons for PE, PP, and PS beads from the Biofilm and Water treatments in relation to their respective controls: **(A)** diameter **(B)** stiffness [*k*], and **(C)** maximum compression [ε_*max*_]. The solid horizontal lines represent the means, error bars represent the 95% confidence intervals, and symbols represent individual observations. Each symbol represents an individual observation. Solid symbols represent experimental treatments (Biofilm or Water) and the hollow symbols of the same color represent their respective controls. The number of observations, *n*, ranges between 8–12, 3–10, and 3–17 for panels **(A–C)** respectively.

There were no significant changes in the mean particle diameter resulting from exposure to either bacterioplankton or sterile water in any of the polymers, as indicated by the comparisons between the untreated and treated MP (two-sample bootstrap test; *p* > 0.2 in all cases; Figure 1D). However, the variance for PE and PS beads in *Biofilm* treatment was significantly lower compared to the respective controls [*F*-test; F_(11, 11)_ = 8.363, *p* < 0.002 and F_(11, 8)_ = 4.301, *p* < 0.05, respectively; Figure 3A]. For any of the polymers tested, no significant differences in the variance were observed between *Water* treatment and untreated controls (*p* > 0.3 in all cases; Figure 1D). Notably, of all polymers tested, PE had the lowest percentage of spherical particles, whereas PS had the highest percentage (Supplementary Table 1), which suggests that it was not the shape but material that promoted the effect. The lower variation in the particle diameter following biofilm formation may indicate that the particle surface becomes more even when bacterial clusters fill cracks and rough patches. However, this explanation was not supported by the surface topography analysis by AFM, as no significant change in surface roughness were detected for either PE or PS following the biofilm formation (Supplementary Figures 2, 4). Thus, the reasons for the increased sphericity of the MP colonized by bacteria and the mechanistic role of biofilms in this change are not clear.

#### Stiffness

Across the polymers, PS was the stiffest material, and PE was the least stiff (Figure 3B). The *Biofilm* exposure significantly reduced the PP stiffness by an average of 35 N/mm (two-sample bootstrap test; *p* < 0.03; Figure 1B), whereas no significant change in the other polymers from the *Biofilm* treatment or any polymers from the *Water* treatment were detected (*p* > 0.25 in all cases). In general, incubation in water is known to soften polymers (Baschek et al., 1999); however, in the PP *Water* samples, stiffness increased slightly but not significantly compared to the control (*p* > 0.2; Figure 1B). Therefore, the reduction in PP *Biofilm* stiffness due to bacterioplankton exposure was not induced by the water exposure alone.

#### Compressibility

Across the polymers, PP and PE had the highest and the lowest maximum compression values, respectively (Figure 3C and Supplementary Figure 5). Moreover, PP samples did not break when subjected to the maximum amount of strain achievable by the instrument (Supplementary Figure 5B); hence, the actual values for PP are likely to be much higher. This precludes a meaningful comparison of maximum compression for PP both within (i.e., control vs. treated) and between (i.e., *Biofilm* vs. *Water*) treatments. However, plastic deformation was clearly observed in all PP samples, which deviated from the linear region after >0.1 mm/mm strain and never reached a fracturing point (Supplementary Figure 5B). The linear region in this curve represents the elastic deformation of the sample, with the plateau arising from the plastic deformation and the densification of the material manifested as the exponential load increase >0.4 mm/mm. This suggests that both PP *Control* and *Biofilm* are ductile, not brittle, and could be quite resistant to breakdown from physical forces, such as waves and abrasion.

There was a significant increase of the PS ε_*max*_ in *Biofilm* treatment compared to that in *Control* (two-sample bootstrap test; *p* < 0.02; Figure 1C), which suggests that the exposed PS were more resistant to breaking down. The load-strain curves for PS beads were linear over most of the strain range before fracturing at 0.16–0.19 mm/mm strain (Supplementary Figure 5C), which would be expected for a brittle material unable to restructure under a stress load. As *Water* treatment had no significant effect on PS ε_*max*_ values (*p* > 0.9; Figure 1C), the observed ε_*max*_ increase in PS *Biofilm* was not likely due to the water exposure alone.

There were no significant differences in the ε_*max*_ values for PE treatments (*Biofilm* vs. *Water*) compared to their controls (*p* > 0.15 in both cases; Figure 1C). A loss in PE tensile strength after colonization by marine bacteria has been reported when exposure lasted over a year, with the earliest measurement taken after 3 months of incubation (Sudhakar et al., 2008). Similarly, Nowak et al. (2011) observed a 33–38% decrease in the tensile strength of PE films due to biodegradation after 225 days of exposure. The strain-load proportional region for PE samples was <0.02 mm/mm, which is higher compared to <0.1 mm/mm for PP and PS. The load-strain curves of PE show that all but two of the control samples fractured, as indicated by a sharp decline and termination of the curve, below 0.04 mm/mm of strain (Supplementary Figure 5A). The PE *Control* samples that did not fracture deviated from the linear proportionality region, where it enters a plastic deformation phase before fracturing. This is an indication of molecular rearrangement to a new equilibrium, suggesting that some of the PE *Control* samples were more ductile compared to the PE *Biofilm* samples, which could be due to the increased degree of crystallinity observed for this material (section Degree of Crystallinity).

### Surface Chemistry

The surface chemistry of PP and PS samples changed significantly in *Biofilm* treatments compared to the respective controls. For both polymers, in *Water* treatment no consistent effects that were different from those of *Biofilm* treatment were observed. In contrast, no measurable effects were induced by the exposure to bacterioplankton in PE.

**Figure 4:**
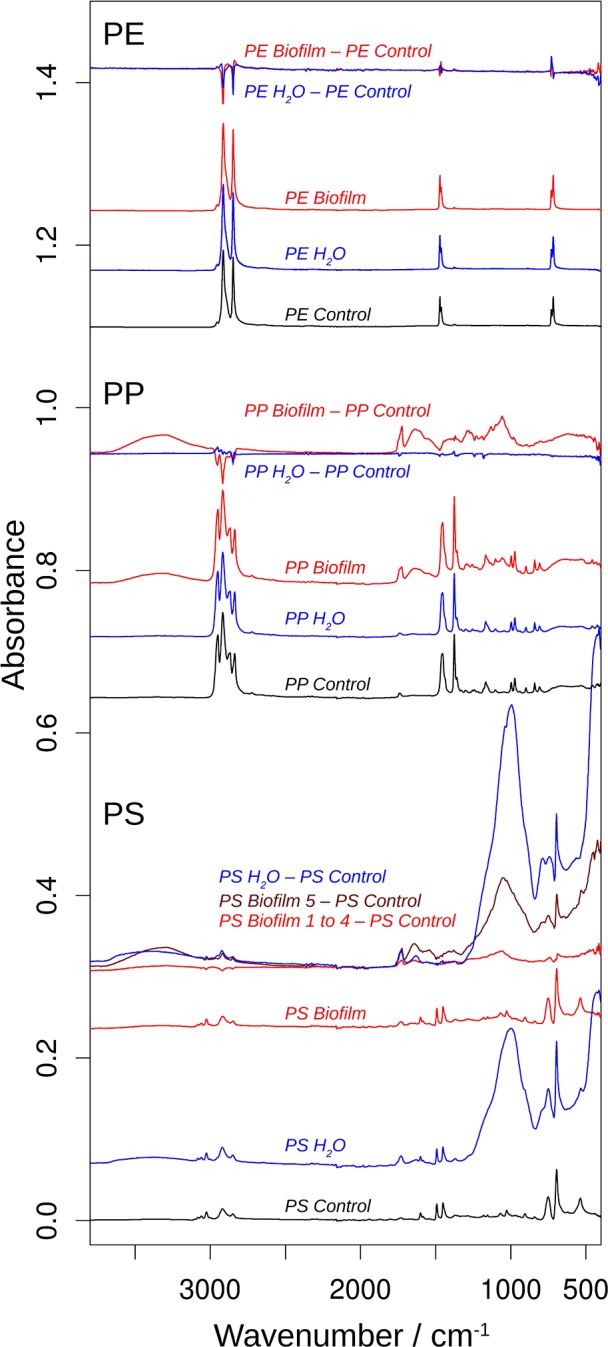
ATR-FTIR spectra of beads of polyethylene (PE), polypropylene (PP), and polystyrene (PP). For each polymer, the bottom three spectra show the spectra of the MP control (black), the MP *Water* (blue), and the MP *Biofilm* samples (red). These spectra were approximately normalized on the main polymer bands by dividing the original spectra by 5 for all PE spectra, by 1.8 for the PP *Control* spectrum, and by 2 for the PP *Water* and PS *Control* spectra. The top spectra for each polymer illustrate the spectral changes that are induced by incubation in seawater and sterilized water. They are subtractions of the respective control spectrum from the MP *Water* (blue) and the MP *Biofilm* (red) spectra. These spectra were multiplied by 2 for a clearer presentation. The dark red difference spectrum labeled *PS Biofilm 5—PS Control* was calculated from a PS *Biofilm* spectrum that significantly deviated from the other four PS *Biofilm* spectra and which therefore was not included in the averaged PS *Biofilm* spectrum and in the difference spectrum labeled PS *Biofilm* 1 to 4—PS *Control*.

**Figure 5:**
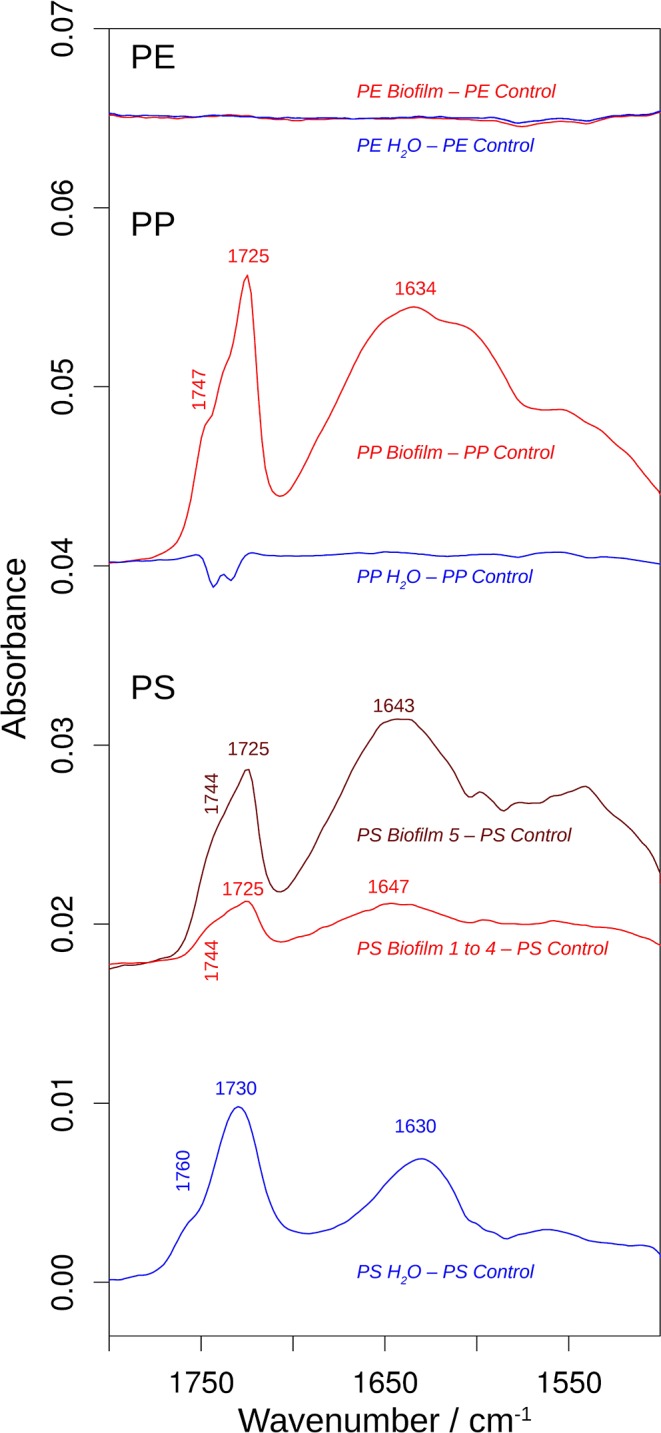
The effects of incubation on the 1,800–1,500 cm^−1^ spectral region of the infrared spectrum. The spectra are the difference spectra shown in Figure 4, but before multiplication by 2. See that figure for more information. The spectral position of the shoulder above 1,740 cm^−1^ was determined from the second derivative spectrum.

Figure 4 shows the obtained spectra of the *Biofilm, Water*, and *Control* samples in all polymers tested, and Figure 5 shows an expanded view of the difference spectra in the 1,800–1,500 cm^−1^ spectral region. If not mentioned otherwise, these spectra were averaged from several measurements (Supplementary Figure 6). The top spectra for each polymer are difference spectra, where the *Control* spectrum had been subtracted from the *Biofilm* and *Water* spectra; these are termed *Biofilm*—*Control* and *Water*—*Control* spectra, respectively (Figures 4, 5). Note that the polymer signals could never be perfectly subtracted in the entire spectral region, which results in positive or negative polymer bands in the difference spectra due to under- or over-subtraction.

The PE spectra did not show significant differences between *Control, Water*, and *Biofilm* samples (top spectra; Figures 4, 5), either because the surface was not modified by the treatments, or because the spectra reflect mainly the absorption of the bulk material. Whereas, analysis employing ATR-FTIR was fully applicable for the PP and PS because the beads remained intact when pressed on the ATR diamond surface, the PE samples were extremely brittle, as demonstrated by the compression test (Supplementary Figure 4A), so the beads crumbled under the force of the ATR stamp even when the force was adjusted carefully. Therefore, our PE spectra mainly reflect the bulk properties of the material, and lack of the measurable effects induced by the exposure to bacterioplankton in PE may, at least in part, be related to these difficulties in obtaining a surface-characteristic spectrum.

In contrast, the surface chemistry of PP and PS *Biofilm* samples and/or *Water* sample was noticeably different from that of the respective *Control* samples. The PP *Biofilm—Control* spectrum shows broad bands around 3,350–600 cm^−1^ (middle series of spectra in Figure 4). The former band is absent in all individually measured *Control* spectra, the difference is therefore significant and the band can be assigned to OH or NH stretching vibrations. The band around 600 cm^−1^ is present to varying degrees also in the *Control* spectra and might therefore not indicate a significantly altered surface chemistry. In addition to the broad bands, the PP *Biofilm—Control* spectrum exhibited distinct bands at 1,725, 1,634, near 1,280, at 1,229, 1,190, 1,133, and 1,058 cm^−1^ (Figures 4, 5). In contrast to the PP beads exposed to bacterioplankton, those exposed to sterilized water did not show significant spectral deviations from the *Control* samples (see the PP *Water*—*Control* spectra in Figures 4, 5).

For PS, the most prominent deviations from the *Control* spectrum were observed for the treatment in sterilized water. The *Water*—*Control* spectrum exhibited broad bands around 3,400 and 1,000 cm^−1^ and distinct bands at 1,730 and 1,630 cm^−1^. In addition, *Biofilm* treatment influenced the surface chemistry of the PS beads. Of the five surface areas probed, four had very similar spectra (Supplementary Figure 5), and were averaged to represent the *Biofilm* spectrum and used to calculate the *Biofilm 1–4—Control* spectrum (Figures 4, 5). This spectrum indicates significant deviations at 1,725, near 1,650 and around 1,060 cm^−1^. Regarding the 1,725 cm^−1^ band, it should be noted that the *Control* spectrum also had a band in this region, but it was considerably weaker; moreover, it was positioned 4 cm^−1^ higher than in the *Biofilm* spectrum. Therefore, we concluded that the *Biofilm* treatment induced a significant spectral change at 1,725 cm^−1^. The spectrum of the fifth surface area probed deviated more from the *Control* spectrum than the other areas. Its difference spectrum was termed *Biofilm 5—Control* spectrum (Figures 4, 5). Upon closer examination, this spectrum was found to be an enlarged version of the difference spectrum obtained with the other four surface areas as it also has bands at 1,725, 1,643, and around 1,050 cm^−1^. Moreover, we recorded additional changes around 3,300 (Figure 4) and 1,540 cm^−1^ (Figure 5) that were less obvious in the averaged spectrum from the other four surface areas. The differences between control and treatment samples observed for the *Biofilm* treatment were different from those of the *Water* treatment. The most obvious difference was the much stronger band near 1,000 cm^−1^ for the *Water* treatment. However, differences were also observed in the 1,800–1,600 cm^−1^ spectral range, where the bands appeared at different spectral positions, thus suggesting alterations in the surface chemistry.

The spectral changes were observed around 3,400, 1,730, 1,640, and 1,000 cm^−1^. They may either be attributed to a degradation of the polymer material or to adsorbed biomaterial, since similar spectral changes are expected in both cases. It is not possible to distinguish with certainty between these possibilities, but the following lines of reasoning indicate a dominance of the polymer degradation processes, at least in the 1,800–1,600 cm^−1^ spectral region:

- Phospholipids of biological membranes typically have their ester C=O absorption near 1,740 cm^−1^ (Tamm and Tatulian, 1997; Naumann, 2001). The main band of *Biofilm*—*Control* (PP and PS) and *Water*—*Control* (PS) spectra in that region was found at lower wavenumber (at or below 1,730 cm^−1^; Figure 4). Therefore, we do not assign it to lipids. The high wavenumber shoulder in the *Biofilm*—*Control* spectra, however, might stem from lipids, but is also commonly observed for degraded polymers as described below.
- Proteins do not seem to contribute substantially to the induced spectral changes. While the band near 1,640 cm^−1^ could be assigned to amide I absorption, a distinct band of somewhat smaller intensity (near 1,550 cm^−1^) should then be expected for the amide II absorption (Rahmelow et al., 1998; Goormaghtigh et al., 2009). Such a band was not detected in our spectra. Also, amide A band near 3,300 cm^−1^ (Sevinc et al., 2015; Nunes, 2016) and Tyrosine shoulder near 1,515 cm^−1^ (Venyaminov and Kalnin, 1990; Goormaghtigh et al., 1994; Barth, 2000), the latter is characteristic band typical for second derivative spectra of proteins, were not apparent in our spectra.

This is why we suggest that PP and PS started to undergo degradation upon exposure to bacterioplankton, whereas some changes were even observed in PS exposed to bacteria-free water. This degradation or possibly migration of additives lead to the appearance of vinyl groups and various oxygenated carbon compounds (Andreassen, 1999; Restrepo-Flórez et al., 2014). In line with this, the treatment-induced spectral changes near 3,400, 1,730, 1,640, and 1,000 cm^−1^ can be assigned to OH, C=O, C=C, and C-O groups (Socrates, 2001; Colthup et al., 2010). In the C=O spectral region between 1,800 and 1,700 cm^−1^, the spectral positions of the maxima determined from the second derivatives between 1,725 and 1,722 cm^−1^ indicate a predominance of ketones in the degradation products (Geuskens and Kabamba, 1982; Lacoste et al., 1993; Socrates, 2001; Colthup et al., 2010). Only in PS *Water* samples a somewhat higher band position (1,729 cm^−1^) was observed, which neither supports nor excludes an assignment to keto groups. The biofilm formation on PP and PS also gave rise to shoulders between 1,747 and 1,738 cm^−1^, which can be assigned to esters. In contrast, exposure of these polymers to sterile water generated shoulders at higher wavenumbers (1,760–1,754 cm^−1^) which could be due to peroxy compounds (Lacoste et al., 1993; Socrates, 2001; Colthup et al., 2010) and carboxylic acids (Philippart et al., 1999). Formation of aldehydes was unlikely, because the typical aldehyde C-H band in the 2,730–2,695 cm^−1^ range (Adams, 1970; Socrates, 2001; Colthup et al., 2010) was absent in our spectra. Likewise, the carboxylic acid species absorbing near 1,710 cm-1 (Adams, 1970; Geuskens and Kabamba, 1982; Lacoste et al., 1993) was not detected.

Degradation-induced changes near 3,400, 1,730, and 1,640 cm^−1^ have been reported for PP (Adams, 1970; Geuskens and Kabamba, 1982; Lacoste et al., 1993; Philippart et al., 1999; Cooper and Corcoran, 2010; Xiong et al., 2017; Auta et al., 2018) and PS (Syranidou et al., 2017) but one should keep in mind that spectral pattern and, therefore, the degradation products are condition-dependent. Isotactic PP that was exposed at different geographic locations showed vinyl bands near 1,630 cm^−1^, carbonyl bands with maxima between 1,730 and 1,720 cm^−1^, and a shoulder at higher wavenumbers (Xiong et al., 2017). Photo-oxidation of PP outdoors and in the laboratory produced a carbonyl band with several shoulders mainly on the high wavenumber side of the band maximum (Philippart et al., 1999). Moreover, the band shape and position—and thus the nature of the carbonyl compounds—depended on light intensity, irradiation time and temperature. The reported maximum was at 1,735 or 1,712 cm^−1^, which is different from the band positions observed in our study, which might be related to differences in both biotic and abiotic conditions. In fact, exposure to polymer-degrading microbes reduced at least some of the spectral changes caused by polymer degradation in PP (Auta et al., 2018) and PS (Syranidou et al., 2017). Moreover, in the latter study a broad band in the 1,200–900 cm^−1^ spectral range for one the of the weathered PS samples was reported. Such a band is particularly prominent in our PS *Water* spectra but also visible in the *Biofilm* samples of PS and PP. In addition to this band, Syranidou et al. (2017) observed a broad band between 1,500 and 1,350 cm^−1^ for one of the weathered PS samples, which was absent in our samples. Taken together, our results indicate surface modifications of the PP and PS *Biofilm* samples that differ from those observed for the respective *Water* samples. These modifications are similar to those reported in previous PP and PS degradation studies.

### Polymers Properties and Bacterial Communities

**Figure 6:**
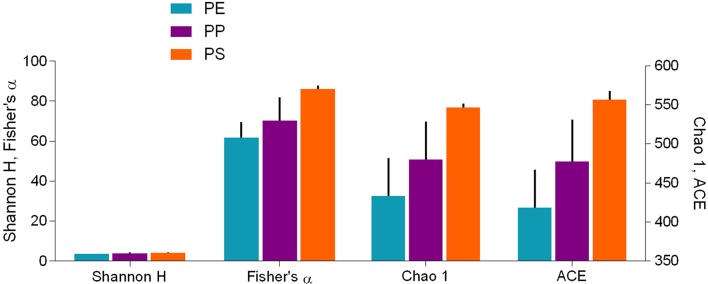
Alpha diversity indices (mean ± SD, *n* = 3) for bacterial communities in the biofilm grown on the test polymers (PE, polyethylene; PP, polypropylene; and PS, polystyrene). Shannon-Wiener (Shannon H) and Fisher's alpha indices are shown on the left y-axis and Chao 1 and ACE estimators are shown on the right y-axis. Statistical comparisons are presented in Table 1; the differences between the polymers were significant (PE vs. PS and PP vs. PS) for Chao1 and ACE but not for the other two indices.

Alpha diversity indices for bacterial communities reflected differences in community composition among the biofilms formed on the test polymers (Figure 6). Moreover, the rarefaction analysis indicated significant differences in taxonomic richness among all polymers (PS > PP > PE; Supplementary Figure 7). All indices suggested that PS communities were also more diverse compared to those in PE and PP. The differences were significant for Chao 1 and ACE, which account for unobserved taxa based on low-abundance features, but not for Shannon-Wiener and Fisher's alpha indices (Table 1) that take into account both richness and evenness and assume that all taxa were represented in the samples (Supplementary Table 2).

Table 1Comparison of the alpha diversity indices (Shannon-Wiener, Fisher's alpha, Chao 1 estimator, and ACE estimator) for bacterial communities identified in the biofilms on the test polymers (PE, polyethylene; PP, polypropylene; and PS, polystyrene): **(A)** ANOVA output (*n* = 3), **(B)** Tukey's multiple comparisons test for the pair-wise comparisons between the indices.

**Table:** 

**(A) ANOVA RESULTS**	**SS**	**DF**	**MS**	**F**	***p*-value**
Interaction	20,458	6	3,410	3.954	0.0069
Polymer	29,092	2	14,546	16.87	<0.0001
Diversity index	1,821,000	3	607,005	703.9	<0.0001
Residual	20,695	24	862.3

**Table:** 

**(B) TUKEY'S MULTIPLE COMPARISONS TEST**	**Mean Diff**.	**95% CI of diff**.	**Summary**
Shannon-Wiener			
PE vs. PP	-0.2906	−60.17 to 59.58	*ns*
PE vs. PS	-0.5423	−60.42 to 59.33	*ns*
PP vs. PS	-0.2517	−60.13 to 59.62	*ns*
Fisher's alpha			
PE vs. PP	-8.404	−68.28 to 51.47	*ns*
PE vs. PS	-24.26	−84.14 to 35.62	*ns*
PP vs. PS	-15.86	−75.73 to 44.02	*ns*
Chao 1			
PE vs. PP	-46.84	−106.7 to 13.03	*ns*
PE vs. PS	-113.7	−173.6 to −53.83	***
PP vs. PS	-66.86	−126.7 to −6.986	*
ACE			
PE vs. PP	-59.39	−119.3 to 0.4814	*ns*
PE vs. PS	-138.7	−198.6 to −78.80	****
PP vs. PS	-79.28	−139.2 to −19.41	**

See Supplementary Table 2 for a short presentation of the diversity indices. Statistical significance in the summary refers to ns: not significant; *p < 0.05; **p < 0.01; ***p < 0.001; and ****p < 0.0001.

Across the polymer types, the Fisher's alpha, Chao 1 and ACE values were significantly negatively related to substrate crystallinity and positively to stiffness and roughness (Table 2; Supplementary Figure 8). No significant correlations for Shannon H were found. The correlations to both crystallinity and stiffness can, at least in part, be related to the fact that these variables significantly correlated with each other (Supplementary Figure 3). However, there were no significant correlations between either of these variables and roughness, which implies that diversity-to-roughness correlation was not driven by the multicollinearity with the other properties.

**Table 2:** Summary of the Spearman's coefficient (ρ) values and their respective significances (*p-*values) for the association between the biofilm diversity metrics and physicochemical properties of the microplastic used as a substrate.

**Physicochemical properties**	**Shannon-Wiener**	**Fisher's alpha**	**Chao 1**	**ACE**
Particle diameter, *d*	−0.316 (>0.4)	−0.580 (>0.1)	−0.580 (>0.1)	−0.580 (>0.1)
Maximum compression, *ε_*max*_*	0.316 (>0.4)	0.263 (>0.5)	0.263 (>0.5)	0.263 (>0.5)
Stiffness, *k*	0.632 (>0.09)	**0.843 (<0.01)**	**0.843 (<0.01)**	**0.843 (<0.01)**
Degree of crystallinity, *X_*c*_*	−0.632 (>0.09)	–**0.843 (<0.01)**	–**0.843 (<0.01)**	**−0.843 (<0.01)**
Surface roughness, *R_*a*_*	0.632 (>0.09)	**0.843 (<0.01)**	**0.843 (<0.01)**	**0.843 (<0.01)**

*Significant correlations are in bold. See Supplementary Table 2 for a short description of the diversity indices*.

Differential abundance analysis identified a total of 7 unique OTUs that were significantly more abundant on one or more polymers (Table 3; Supplementary Figure 9); on average, a 4.1 log_2_ fold-change in the differentially abundant OTUs was observed. The differences were mainly related to the PE samples that had a significantly higher relative abundance of *Sphingobium, Novosphingobium* and uncultured Planctomycetaceae (four OTUs) compared to the PP and PS samples. The latter two polymers had a significantly higher abundance of uncultured Sphingobacteriales and Alphaproteobacteria (three OTUs) compared to PE communities.

**Table 3:** OTUs with closest taxonomic identification for bacterial groups that had significantly higher relative abundance on specific test polymers.

**OTU**	**Taxonomic identification**	***p*-value**	**FDR**
*OTU_000291*	Proteobacteria, Alphaproteobacteria, Alphaproteobacteria incertae Sedis (uncultured)	1.73E−05	0.00685
*OTU_000164*	Bacteroidetes, Sphingobacteriia, Sphingobacteriales (uncultured)	3.33E−05	0.00685
*OTU_000139*	Bacteroidetes, Sphingobacteriia, Sphingobacteriales, Saprospiraceae (uncultured)	3.87E−05	0.00685
**OTU_000084**	Proteobacteria, Alphaproteobacteria, Sphingomonadales, Sphingomonadaceae, *Novosphingobium*	4.14E−05	0.00685
**OTU_000037**	Planctomycetes, Planctomycetacia, Planctomycetales, Planctomycetaceae (uncultured)	0.000287	0.037723
**OTU_015077**	Proteobacteria, Alphaproteobacteria, Sphingomonadales, Sphingomonadaceae, *Sphingobium*	0.000342	0.037723
**OTU_001040**	Proteobacteria, Alphaproteobacteria, Sphingomonadales, Sphingomonadaceae, *Sphingobium*	0.000516	0.048791

*OTUs that were significantly higher in PE are in bold, and those that were significantly higher in PP and PS are in Italics. To identify as many significant comparisons as possible while still maintaining a low false positive rate, the False Discovery Rate (FDR) at level α = 0.05 were applied*.

There is a little understanding of how polymer crystallinity impacts biofilm composition and diversity on the polymer surface under environmental conditions. The MP with the lowest microbial diversity and approximately 5-fold prevalence of *Sphingobium* (OTU_001040 and OTU_015077), 3-fold prevalence of *Novosphingobium* (OTU_000084), and 2-fold prevalence of uncultured Planctomycetaceae (OTU_000037) was PE; this was also the polymer with the highest degree of crystallinity and the lowest stiffness and roughness among the polymers tested (Figures 2, 3). Furthermore, PE was the only MP that experienced a significant increase in crystallinity following the biofilm formation, suggesting degradation of the amorphous regions during the incubation. If the microorganisms colonizing PE surfaces were able to utilize these amorphous components of the polymer as a carbon source, this could have be a selective force favoring such taxa and lowering the overall microbial diversity. For such communities, particularly strong effects would be observed for indicators placing weight on the unobserved and rare taxa, such as Chao 1 and ACE, and this is what we found (Table 1).

Both genera *Sphingobium* and *Novosphingobium* have taxa with high capacity for polymer degradation, particularly polymers like polyvinyl alcohol (PVA), i.e., polymers with low polymerization and high saponification (Pathak and Navneet, 2017). Therefore, their preferential occurrence on PE is in line with the utilization of the amorphous regions as a carbon source. Similarly, in line with our findings, several studies using different experimental approaches and from different locations reported Planctomycetes to be significantly more abundant on PE. Moreover, this is a family rich in taxa that are overrepresented in *plastisphere* associated to diverse plastics and involved in biodegradation (Kirstein et al., 2019). These prokaryotes are particularly abundant in marine surface waters, where they are often associated with marine snow and degradation of complex carbon substrates and high molecular weight compounds, including polymers, e.g., sulfated polysaccharides (Wiegand et al., 2018).

Several studies have found a correlation between a substrate stiffness and biofilm formation. In general, the harder the surface, i.e., greater moduli and stiffness, the greater bacterial adhesion (Lichter et al., 2008). As a result, selection on such surfaces is weaker and biofilm formation is quick with more diverse composition (Saha et al., 2013; Guégan et al., 2014; this study). However, an inverse relationship between cell adhesion and moduli has also been observed, with scarcer biofilms on harder surfaces. Substrate stiffness has also been suggested to influence protein synthesis in biofilms (Guégan et al., 2014). Although the mechanisms for the latter remain unclear, substantial evidence points to bacteria being able to sense and respond to surface hardness (Song et al., 2018).

Higher surface roughness implies greater habitable area (Anselme et al., 2010), which has been demonstrated experimentally (Bohinc et al., 2014; Yoda et al., 2014). However, surface modifications on the nanoscale can also lead to a decrease in biofilm formation due to the relatively large size of a common bacteria cell in relation to the surface indents (Seddiki et al., 2014). In addition to facilitation of attachment, surface roughness can also lead to physiological changes to the bacteria that colonize it, such as loss of flagella and concomitant decrease in motility (Singh et al., 2013). We found that rougher surfaces were inhabited by more diverse biofilms, which was mostly reflected in the highest diversity observed in PS-associated biofilms (Supplementary Figure 8). Moreover, uncultured Sphingobacteriales that was overrepresented on PP and PS surfaces (Table 2; Supplementary Figure 9) are hydrocarbonoclastic bacteria reported for *plastisphere* communities (Kirstein et al., 2019).

## Conclusions

During a 2 week exposure to ambient bacterioplankton, MPs underwent physicochemical changes, which varied with regard to both physicochemical parameters and polymers. The most significant changes were: increase in crystallinity (PE); decrease in stiffness (PP); and increase in the maximum compression (PS). These changes were not observed when MPs were incubated in bacteria-free water, which implies that microorganisms of the biofilm communities were involved in the observed physical perturbations. Furthermore, in virgin polymers, some physicochemical properties were significantly cross-correlated (i.e., stiffness~crystallinity, stiffness~roughness, and crystallinity~roughness). However, upon biofilm formation, the correlations between stiffness~roughness and crystallinity~roughness were lost. Therefore, biofilm-mediated weathering caused physicochemical properties to converge over time. The exact mechanisms of these environmental transformations remain to be studied, but they are most likely involve combined effects of microbial attachment and biodegradation, as well as water absorption into the polymer matrix. However, it cannot be ruled out that the observed changes in the polymers were due to the biological degradation of specific additives that might have been present in the test microplastics. We also found that substrate properties may affect (or select for) biofilm communities, e.g., higher diversity of bacteria were observed on the substrates with low crystallinity, but higher stiffness and surface roughness. Notably, these significant changes occurred rapidly and in the absence of physical forces, such as UV light and shear forces.

## Data Availability Statement

The datasets generated and codes used for analysis can be found in the following repository: https://github.com/emcgi/MPbiofilm.

## Author Contributions

EG, EM, and MO designed the research. EM and EG wrote the paper and analyzed the data. LC and MH performed DSC, compression, SEM, and AFM analysis and interpreted their results. AB and EH-B performed FTIR and interpreted the results. EG performed microbial community analysis. All authors contributed to the writing.

### Conflict of Interest

The authors declare that the research was conducted in the absence of any commercial or financial relationships that could be construed as a potential conflict of interest.
