# Supplementary material for: Rapid Physicochemical Changes in Microplastic Induced by Biofilm Formation
Source: Front Bioeng Biotechnol. 2020 Mar 20;8:205. doi: 10.3389/fbioe.2020.00205 (PMC7103643; doi:10.3389/fbioe.2020.00205)
Supplement: Supplementary file 1 [file Data_Sheet_1.PDF]

Supplementary Material

Table S1. Nominal characteristics of the microplastics used in this study as specified by the manufacturer; modified from Ogonowski et al. 2018. All particles are precision spheres with nearly spherical shape; all were purchased from Cospheric (Santa Barbara, CA); www.cospheric.com

Polymer	Product ID	Spherical particles	Particle diameter, mm	Density, g/cc	Specific surface area, mm^2/g	Water contact angle, Θ °
Clear polyethylene; PE	CPB-0.96	>75%	2.0-2.4	0.96	3.2	101.7
White polypropylene; PP	PPS-0.9	>95%	2.5	0.866	4.6	99
White polystyrene; PS	PSS-1.05	>99%	2.0	1.055	3.2	87

Table S2. Short summary for α diversity metrics applied for bacteria community analysis in the biofilms formed on MP.

Diversity indices	Short description	Reference
Shannon-Wiener diversity index (Shannon H)	Estimator of species richness and species evenness: more weight on species richness. Assumes that all species from a sampled population are represented. Can be difficult to interpret when communities differ significantly in richness.	(Lemos et al., 2011)
Fisher's alpha (α)	A parametric index of diversity that assumes that the abundance of species follows the log series distribution. Used as a diversity index to compare among communities varying in number of individuals, because theoretically independent of sample size. However, alpha may be underestimated in communities in which the spatial arrangement of individuals is strongly clustered, or in which the total number of species does not tend to infinity.	(Fisher et al., 1943)
Chao 1 estimator	Estimates diversity from abundance data. Is based on the concept that rare species infer the most information about the number of missing species. Assumes that number of observations for OTU follows Poisson distribution; correction for variance is applied.	(Chao, 1984)
ACE estimator	Abundance-based coverage estimator of species richness. A nonparametric method for estimating the number of OTUs using sample coverage, which is defined as the sum of the probabilities of the observed OTUs. The estimation of the number of missing species is based entirely on the abundance of rare species.	(Chao and Chiu, 2016)

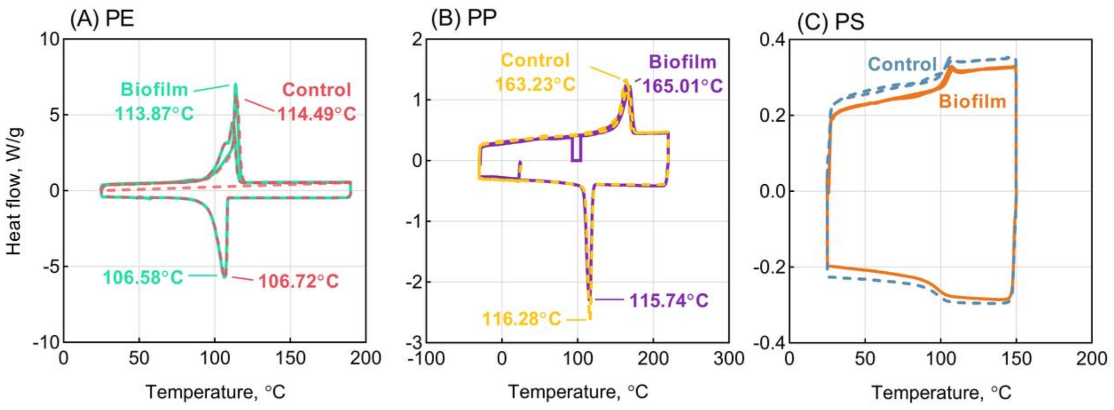

Supplementary Figure S1. Differential scanning calorimetry (DSC) curves for polyethylene (A), polypropylene (B), and polystyrene (PS).

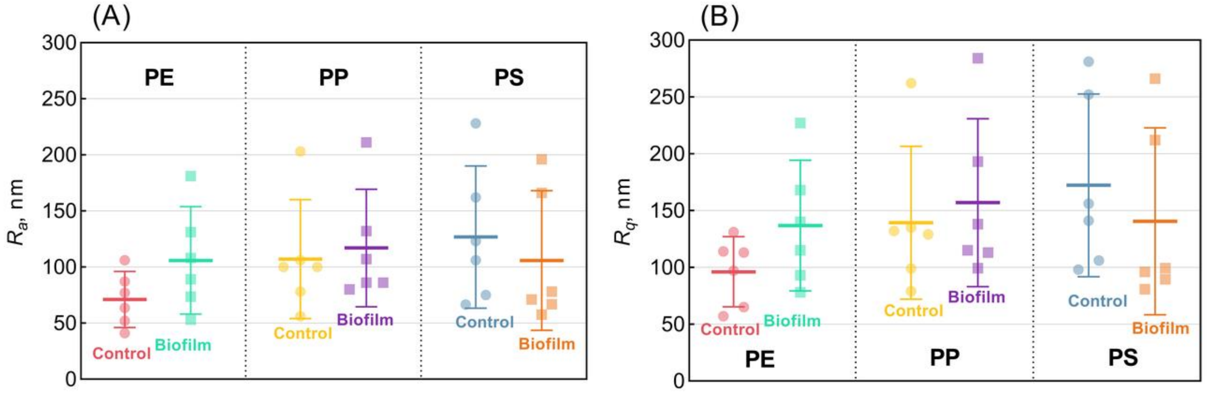

Supplementary Figure S2. Surface topography analysis by AFM. Surface roughness in the polymers from *Biofilm* treatment and the untreated controls calculated using AFM measurements: (A) Average arithmetic roughness (R_a) and (B) average root mean square roughness (R_q). The thick horizontal lines indicate the mean value, error bars represent 95% confidence intervals, and the data points show individual replicates (n = 5 to 6). The details on the polymers (PE, PP, and PS) are provided in Supplementary Table S1.

The order of the average surface roughness in the test materials in the controls was PE < PP < PS. The marginally significant difference in R_a values was found between the untreated PE and PS (Bootstrap Hypothesis Testing; $p > 0.06$). However, this difference disappeared after the biofilm formation, as indicated by the comparison of the PE and PS samples from the *Biofilm* treatments ($p > 0.9$). Thus, variability in the surface roughness between the polymer materials may decrease following biofilm formation. These findings also support the outcome of the cross-correlation analysis for the physicochemical variables measured in this study: significant correlations for $k \sim Rq$ and $X_c \sim R_a$ were present in the *Control* treatments but disappeared in the *Biofilm* treatments (Supplementary Figure S3).

While none of the polymers exhibited a significant change in either R_a or R_q values upon the exposure to bacteria (*Biofilm* vs. *Control*; R_a: $p > 0.13$ in all cases; 0.13 för PE, 0.74 för PP, and 0.49 för PS; see also Figure 1), changes in PE surface roughness should be investigated further. Moreover, some limitations with the AFM tip led us to believe that PS roughness calculations should be interpreted with caution. In particular, the length of the AFM needle determines the maximum height difference that the instrument can detect. Therefore, the surface being analyzed cannot have a peak-to-valley difference greater than the needle length, and the cantilever arm will prevent the needle from following the curve of the surface. When analyzing PS, several locations had to be omitted due to this error. Therefore, the roughness of PS presented was likely underestimated in our study.

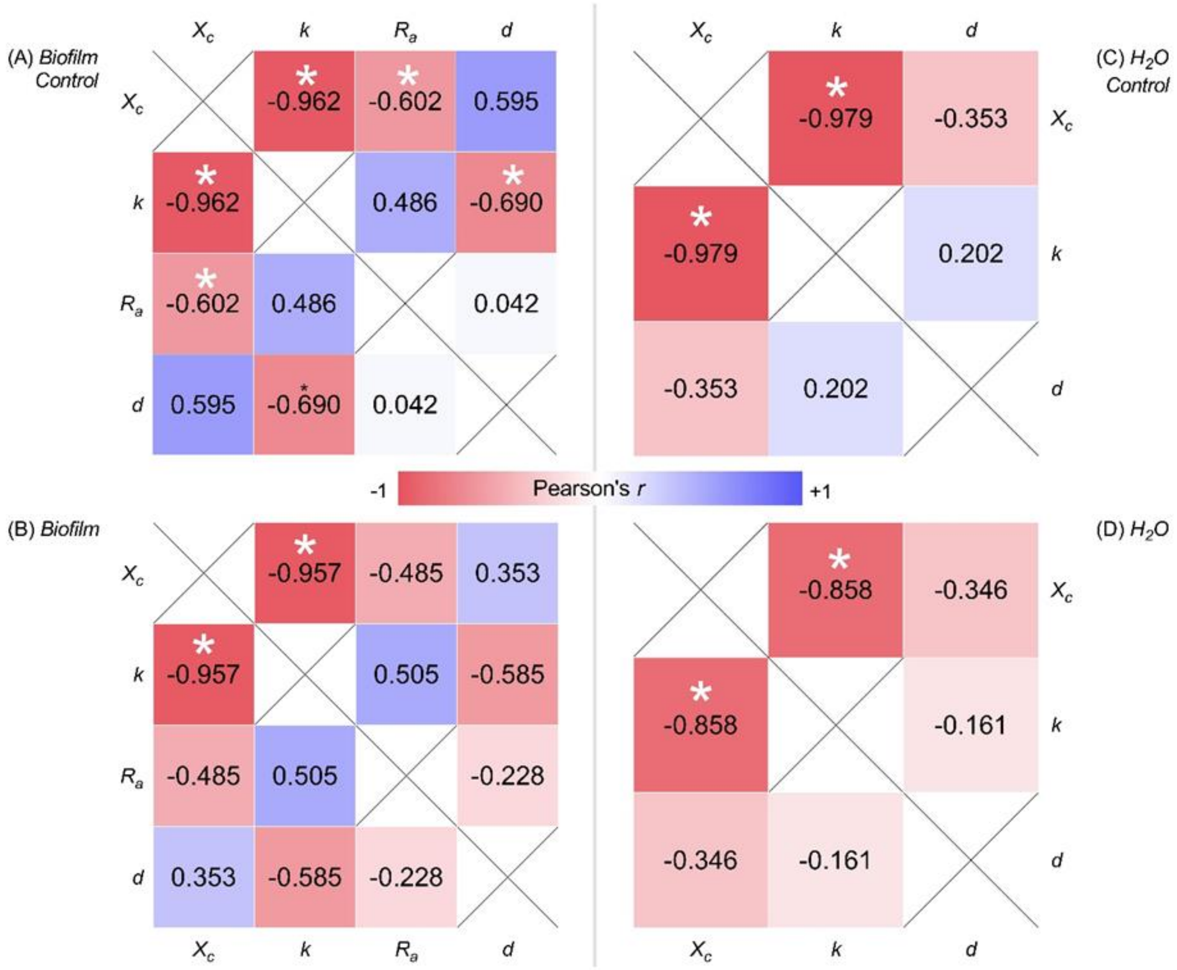

Supplementary Figure S3. Cross-correlation between physiochemical variables. Correlation coefficients (Pearson's *r*) matrices for physiochemical variables of *Biofilm Control* (A), *Biofilm* (B), *H_2O Control* (C), and *H_2O* (C) samples. Variables include crystallinity (X_c), stiffness (*k*), maximum compression (\epsilon_max), arithmetic roughness (R_a), and diameter (*d^hat*). Numbers inside of the boxes represent the calculated Pearson's *r*. * indicates that the 95% confidence intervals do not overlap zero

Several physiochemical properties, across polymer type, were significantly correlated (p<0.05) in the *Biofilm Control* MPs (Supplemental Figure 7A), however, after *Biofilm* treatment, significant correlations for $k \sim d$ and $X_c \sim R_a$ were lost (Supplemental Figure 7B). However, the negative correlation between $k \sim X_c$ remained significant in the *Biofilm* samples (p<0.05). The only significant correlation between variables in *H_2O* treatments, $k \sim X_c$ (Supplemental Figure 7D), was also present in the *H_2O* Control (Supplemental Figure 7C).

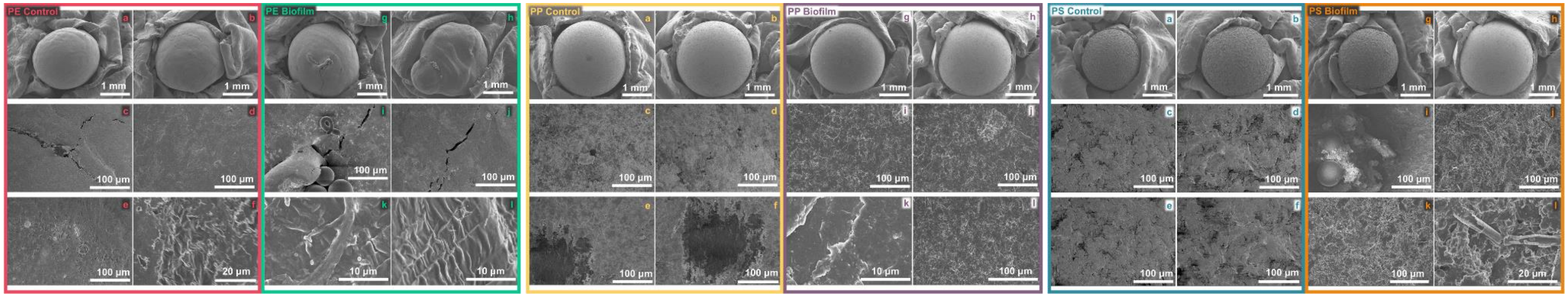

Supplementary Figure S4. SEM images of polyethylene (PE, left panels), polypropylene (PP, middle panels), and polystyrene (PS, right panels); for each material, control and biofilm samples are shown. For each material, the following features are shown: (A-B) untreated beads, (C-F) surface topography such as cracks and ridges of untreated beads, (G-H) beads carrying biofilm, (I-L) surface topography, such as cracks, ridges, and foreign bodies attached to the biofilm-carrying beads. Note that color coding corresponds to that in Supplementary Figure 2S.

Note that PE beads were the least spherical and had visible cracks along the surface, in both *Control* and *Biofilm* samples; this is inagreement with their specifications provided by the manufacturer (Supplementary Table S1). Higher magnification SEM images of PE *Biofilm* show the accumulation of small spheres that seemed to congregate along the cracks and ridges of the surface; small ridge-like and filament-like patterns were also more abundant compared to the PE *Control*. There was little difference between PP *Control* and *Biofilm* surfaces, with less distinct features than was observed in the PE surfaces. Small spherical objects appeared to be aggregating in the crevices on the PS *Biofilm* samples, similar to PE *Biofilm* surfaces. There were also some tubular shaped objects attached to the PS *Biofilm* surfaces that were not found in PS *Control* samples.

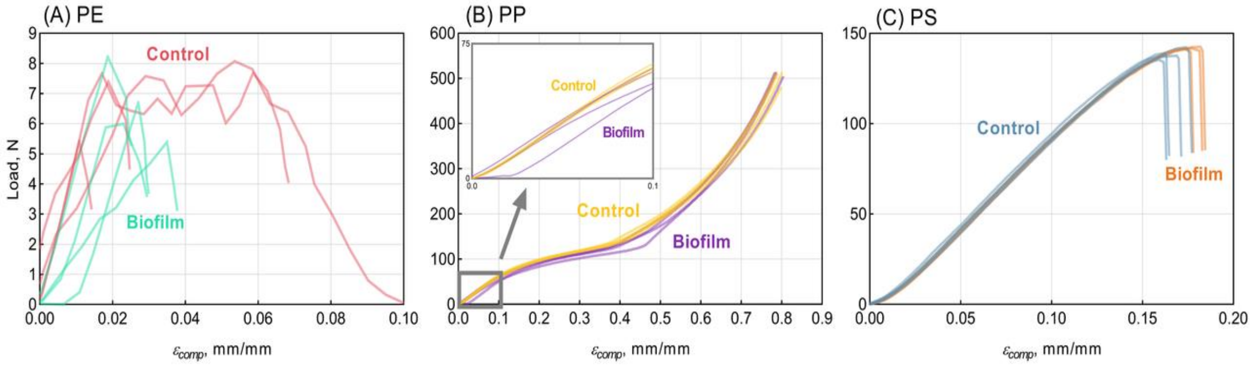

Supplementary Figure S5. Load-compression curves for (A) PE, (B) PP, and (C) PS. Each curve represents one measurement (n = 3 to 5 per group). Biofilm treatment and respective control are shown for each material. Color conding as in Supplementary Figures S1-S3.

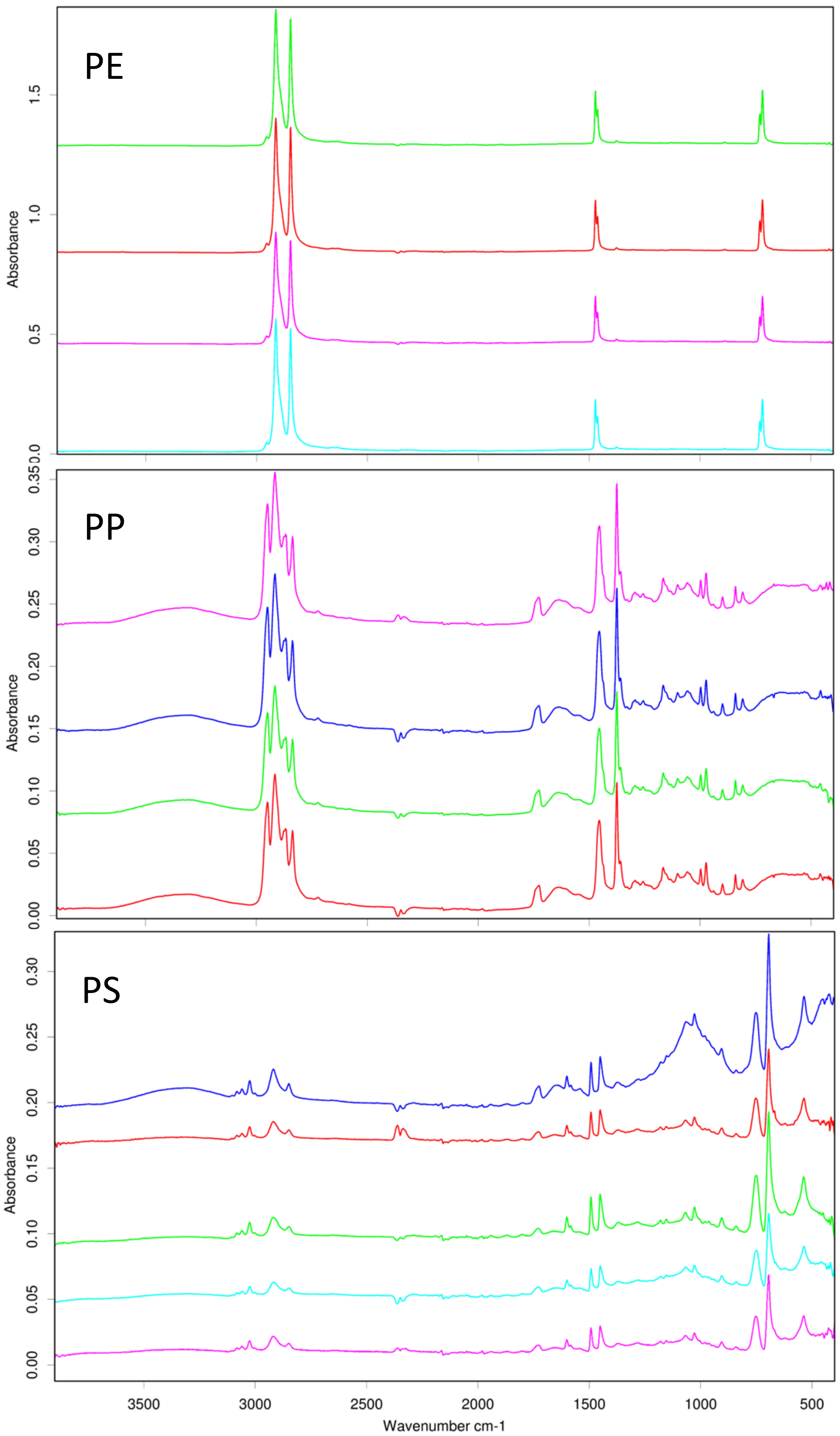

Supplementary Figure S6. ATR-FTIR spectra of samples from *Biofilm* treatments for each polymer tested (n = 4 to 5).

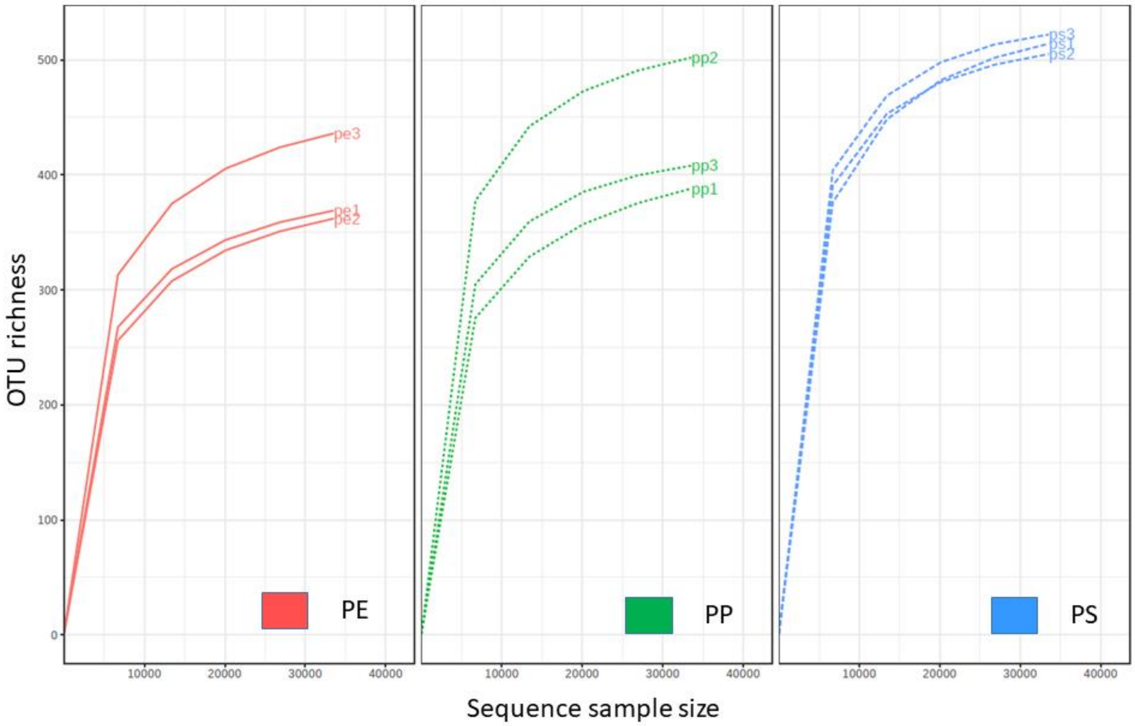

Supplementary Figure S7. Rarefaction curves for OTUs obtained for bacterial communities on the test polymers. Good's coverage was > 99.8 for all samples indicating that the communities were sampled adequately. The highest and lowest OTU richness was observed for polypropylene (PS) and polyethylene (PE), respectively, with significant differences between all polymers (Tukey's multiple comparisons test; PE vs. PP: $p < 0.05$; PE vs. PS: $p < 0.0001$; PP vs. PS: $p < 0.01$). The PS samples had also the lowest within-treatment variability.

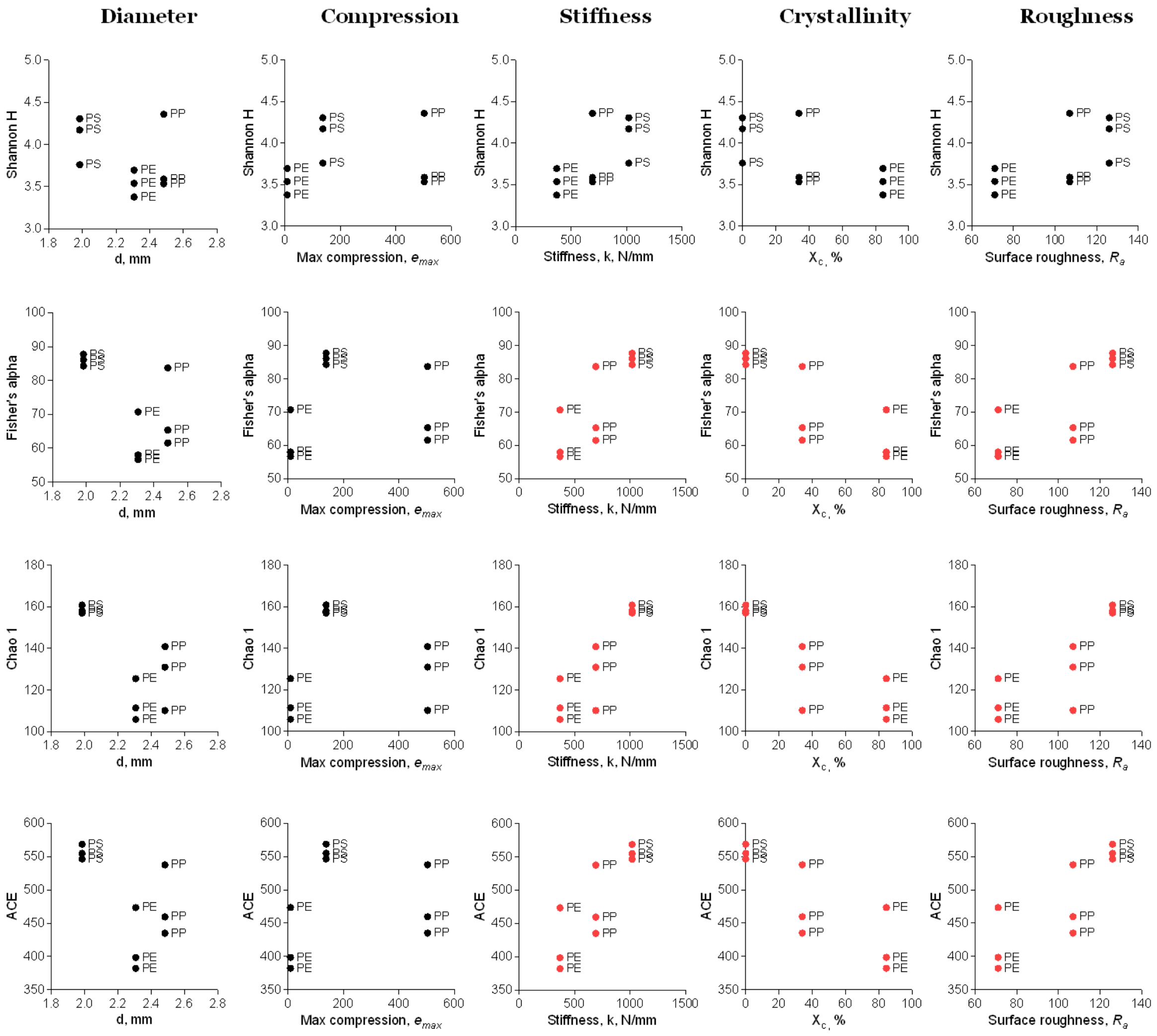

Supplementary Figure S8. Alpha diversity indices versus mean values (within polymer type) of the measured physicochemical properties: quantitative diameter (*d*), maximal compression (ε_{max}), stiffness (*k*), degree of crystallinity (X_c), and arithmetic roughness (R_a). The properties are indicated on the top of the figure, and the data for each property by each diversity metric are presented in vertical direction. Spearman's ρ correlation was calculated for each dataset and the correlations are presented in Table 2. Here, the significant ($p < 0.05$) correlations are shown in red. Polymer type (PE, PP, and PS) are indicated for each data point.

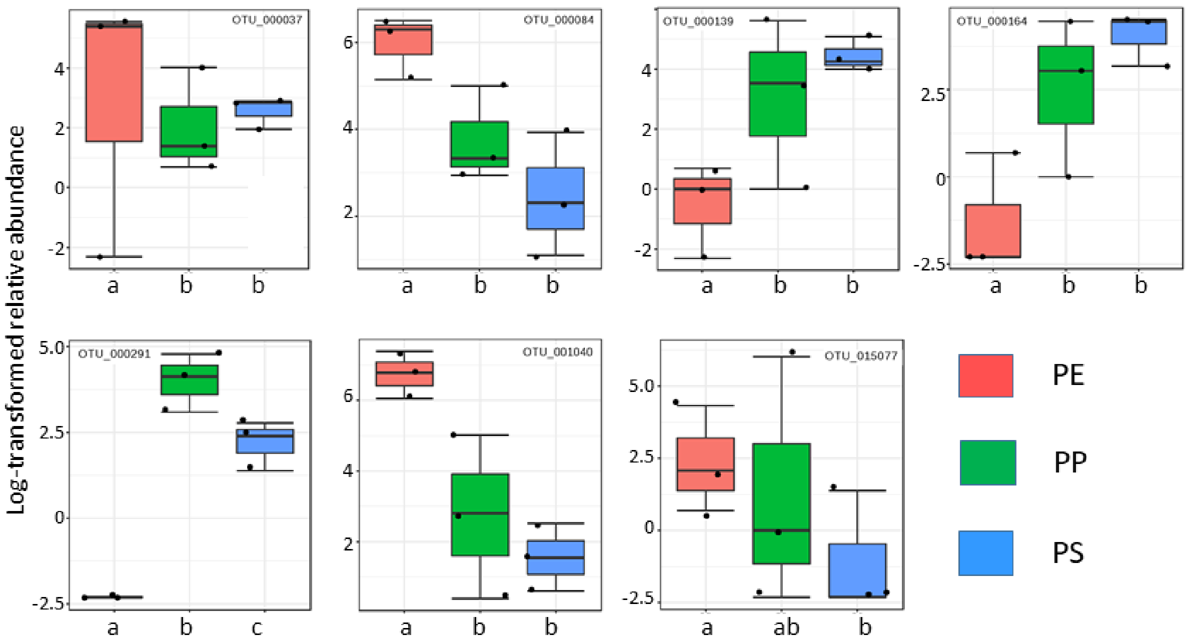

Supplementary Figure S9. Differential abundance of specific OTUs associated with biofilms on the test polymers (PE: polyethylene, PP: polypropylene and PS: polystyrene). The data are shown for OTUs with significantly different log-transformed relative abundance (FDR < 0.05). The non-matching letters along the horizontal axis indicate significant differences between the polymers.

References

Chao, A., 1984. Nonparametric Estimation of the Number of Classes in a Population. Scand. J. Stat. 11, 265–270.

Chao, A., Chiu, C.-H., 2016. Species Richness: Estimation and Comparison, in: Balakrishnan, N., Colton, T., Everitt, B., Piegorsch, W., Ruggeri, F., Teugels, J.L. (Eds.), Wiley StatsRef: Statistics Reference Online. John Wiley & Sons, Ltd, Chichester, UK, pp. 1–26. https://doi.org/10.1002/9781118445112.stat03432.pub2

Fisher, R.A., Corbet, A.S., Williams, C.B., 1943. The Relation Between the Number of Species and the Number of Individuals in a Random Sample of an Animal Population. J. Anim. Ecol. 12, 42. https://doi.org/10.2307/1411

Lemos, L.N., Fulthorpe, R.R., Triplett, E.W., Roesch, L.F.W., 2011. Rethinking microbial diversity analysis in the high throughput sequencing era. J. Microbiol. Methods 86, 42–51. https://doi.org/10.1016/j.mimet.2011.03.014
